# Physical activity in the classroom to prevent childhood obesity: a pilot study in Santiago, Chile

**DOI:** 10.1017/jns.2017.14

**Published:** 2017-05-15

**Authors:** Francisco Mardones, Pilar Arnaiz, Johana Soto-Sánchez, Juana Saavedra, Angélica Domínguez, Jaime Rozowski, Laura Iriarte, Jennifer Cantwell Wood

**Affiliations:** 1 Department of Public Health, Pontificia Universidad Catolica de Chile, Marcoleta 434, Santiago, Chile; 2 Department of Pediatrics, Pontificia Universidad Catolica de Chile, Santiago, Chile; 3 Physical Education, School of Exercise Science and Sport, Universidad de Playa Ancha Facultad de Ciencias de la Educacion, Valparaiso, Chile; 4 Department of Nutrition, Pontificia Universidad Catolica de Chile, Santiago, Chile; 5 ILSI Sur Andino, International Life Sciences Institute (ILSI Sur Andino), Santiago, Chile; 6 Family Nutrition of the Triad, LLC, 1108 Grecade Street, Suite 212, Greensboro, NC 27408, USA

**Keywords:** School age population, Physical activity, Blood pressure, Waist circumference, Behaviour, Appetite, Obesity, ILSI, International Life Sciences Institute

## Abstract

This paper describes a 4-month pilot study that tested the suitability of a physical activity intervention for first graders (children aged 6 and 7 years) in a public school in Santiago, Chile. Teachers were trained to deliver the programme in the classroom during the school day. Teachers were surveyed to determine if this intervention fit within their curriculum and classroom routines and they reported in a focus group that it was suitable for them. All children actively participated in the programme and positive changes in their attitudes towards physical activity were observed by their teachers. Anthropometrics, blood pressure and hand grip strength were measured in the students. A significant reduction was observed in children with high waist circumference ≥ 90th percentile, and in mean systolic blood pressure. However, statistical power values for those comparisons were rather low. Anthropometry and hand grip strength were not modified. The latter calculations and the lack of a control group are showing the weaknesses of this pilot study and that further research with a larger sample size and an experimental design is strongly needed.

Childhood obesity is a growing problem that has evolved into a serious public health crisis in Chile. The obesity rate for elementary school children aged 6–8 years was 25·3 % in 2013, up from 17 % in 2001^(^^1^^)^. Obesity is associated with many negative health outcomes in the general population^(^^2^^)^. In Chile, we have seen an upsurge in the diagnosis of the metabolic syndrome and insulin resistance in children and adolescents^(^^3^^,^^4^^)^. In children with elevated blood pressure and low levels of HDL-cholesterol, we have seen an increase in subclinical atherosclerosis as determined by carotid intima-media thickness testing^(^^5^^)^.

Overweight and obesity derive from an imbalance between energy intake *v*. energy expenditure. One possible way of reversing this imbalance is by increasing energy expenditure through moderate to vigorous physical activity. The WHO recommends at least 60 min of moderate to vigorous intensity physical activity for all children. For children between the ages of 5 and 17 years these activities may include play, games, sports, transportation, chores, recreation, physical education or planned exercise in the context of family, school and community activities^(^^6^^)^. This level of activity has been demonstrated to improve cardiovascular function, respiratory function, muscular function and bone health, and to reduce symptoms of anxiety and depression^(^^7^^)^. Data show that few Chilean children reach these levels of physical activity and 64 % of children in Chile get little, if any, physical activity at all^(^^8^^)^.

One way of increasing physical activity is to include it in the daily classroom routine. In the USA in 1999, the International Life Sciences Institute (ILSI) Research Foundation developed a classroom curriculum for physical activity, which reinforces the regular classroom course of study, called TAKE10!^®^. The curriculum includes directed activity that can be accomplished within the classroom setting in 10-min increments three times per d^(^^9^^)^. An evaluation conducted after 10 years of programme implementation concluded that the programme is a practical way to increase physical activity within the classroom and works to improve focus and learning in students^(^^10^^)^.

This pilot study tested the implementation of the TAKE10!^®^ programme in first-grade students at the Luis Matte Larraín public school in the municipality of Puente Alto in Santiago, Chile. The main objective of the study was to test the suitability of the programme for the students and teachers in the school as a way of increasing any form of physical activity within the classroom. Other changes measured were anthropometrics, blood pressure and physical fitness.

## Methods

This study was implemented in three first-grade classrooms at the Luis Matte Larraín public school in the municipality of Puente Alto in Santiago, Chile. The teachers were trained and followed prospectively for 4 months, August to November, corresponding to winter and spring semesters of 2014. The study included 3 months of active training and intervention. The TAKE10!^®^ programme, originally developed for educators, consists of a set of activity cards and other materials that have short programmes of physical activity for the students to do within the classroom as part of the regular school day^(^^9^^)^. The curriculum activities also incorporate classroom content that is age appropriate and tied to the physical activities. For example, within a mathematics lesson, the teacher can give an addition exercise where the students have to give the answer by jumping the correct number of times.

The principal agent for the initiation of this study was the physical education expert on the research team (J. S.-S.), who was previously trained in the TAKE10!^®^ programme. She gave an informative talk for the school faculty heads where she presented the programme objectives, timeline and expected results. Although the original proposal for the study included incorporating the activities for 10 min three times per d, 5 d per week, it was later decided to apply the model only twice per d. As this school operates on a 5-h school day as opposed to the normal 7-h school day, the teachers felt a time constraint in achieving the activity objectives in addition to their usual academic objectives within this time-frame. Consequently, the time allotted each day for the intervention activities were shorter than originally planned.

The intervention included an extensive training period for the teachers that took three stages over the course of 3 months. During the first month, in order to introduce the programme and train the teachers, our physical education expert went to the school to lead the activities within each of the three classrooms twice per d for 5 d per week. She led the students in 10 min of activity following the first-grade curriculum cards of the TAKE10!^®^ programme, which were translated into Spanish by the ILSI Southern Andes group. The teacher was present for these expert-led activities for the first month of the intervention. In the second month, our physical activity expert shared in the duties of leading the activity with each classroom teacher while the teacher progressively took full responsibility for leading the activity. In the last month, each classroom teacher was responsible for fully implementing all TAKE10!^®^ activities; our physical activity expert supervised by giving performance feedback and supporting the teachers with problem-solving skills.

The materials for the first-grade level included thirty-nine cards with intentional movements related to the established curriculum. It was necessary to adapt some of the programing to the specific local needs of the students in Chile. For example, local Chilean terms were used to modify the examples and instructions given on the cards, making them easier for the children to understand. Also, the lessons were modified to use phonetic sounds as opposed to letters for easier memorisation. A separate article will present an evaluation and explain what adaptations were made to the TAKE10!^®^ programme activities in order to make them useful in this setting.

The suitability of the programme was evaluated through daily observations by one of our co-investigators (J. S.-S.). This investigator determined the level of participation in the activities by the teachers and the students. At the end of the study, we conducted a focus group with the teachers and the school principal to solicit qualitative data about the suitability of the programme.

Evaluation of the potential impact of the programme on the children's health was determined by anthropometric measurements, blood pressure measurements, and hand grip strength evaluation by the study group nutritionist (L. I.). Data were collected both pre- and post-intervention. Height and weight measurements were taken on a beam scale with a stadiometer (SECA^®^ model 700). The children were measured barefoot in their underwear, with the average mass of the clothing subtracted from the measurement. Each measurement was taken three times and averaged^(^^11^^)^. BMI was calculated (kg/m^2^) and was expressed in percentiles referenced to the Centers for Disease Control and Prevention National Center for Health Statistics (CDC-NCHS) 2000 growth charts. Nutrition status was classified as BMI percentile groups: <5, malnutrition, 5–84 normal-weight status, 85–94 overweight, ≥95 obesity^(^^11^^)^. Waist circumference was measured by a non-stretch measuring tape at the right lateral superior point of the iliac crest on the mid-axillary line at the end of an exhalation, according to international standards. Each measurement was taken three times and averaged. All measurements that were ≥ 90 percentile of the reference were classified as high waist circumference^(^^12^^)^. Triceps and subscapular skinfolds were measured with Harpenden calipers and a standard measurement technique. These two measurements were used to calculate body fat percentage according to the Slaughter *et al.* skinfold-thickness equations^(^^13^^)^. Blood pressure was measured with a Dynamap Pro 100 Critikon patient monitor according to international standards. Three measurements were taken on each child and averaged. All average measurements that were ≥90 percentile of the reference were classified as high blood pressure^(^^14^^)^.

Hand grip strength was measured by a hand dynamometer^(^^15^^–^^17^^)^ The measurement was taken by noting the maximum pressure applied to the dynamometer for 2 s according to a standard measurement technique, keeping the arm at the side of the body, with the elbow extended. This measurement was repeated three times on both the right and left arms. The highest value was taken from the three measurements on each arm and the average was taken of the two measurements.

All categorical variables were described with percentages while continuous variables were described by the mean and standard deviation. In order to describe anthropometric changes pre- and post-intervention, we evaluated dependent variables by using Student's *t* test, and McNemar's test for comparison of proportions. All tests were two-tailed, and significance was defined as a *P* value of less than 0·05. For all comparisons with significant differences the power of the statistical test was expected at 0·80. All statistical analyses were performed with SPSS 17.0 software (IBM Corp.).

Informed consent was obtained from the parents of all participating children and the school principal. As there were no physical measurements taken by any teacher, it was not deemed necessary to provide informed consent for their participation. Teachers were given a questionnaire at the beginning and end of the intervention. All study activities were approved by the Scientific Ethics Committee of the School of Medicine, Pontificia Universidad Católica de Chile, Santiago, Chile.

### Ethical approval

This study was conducted according to the guidelines laid down in the Declaration of Helsinki and all procedures involving human subjects were approved by the Scientific Ethics Committee of the School of Medicine, Pontificia Universidad Católica de Chile, Santiago, Chile.

### Informed consent

Informed consent was obtained from all individual participants included in the study. For participants under the age of consent, informed consent was obtained from a parent or legal guardian.

## Results and Discussion

All the teachers followed the stages previously described for their training over the 4 months of the pilot study while being supervised by our physical education expert (J. S.-S.). The TAKE10!^®^ activities were done twice per d, 5 d per week for the 3 months of the active intervention period. All participating students followed the instructions for the activities as reported by our co-investigator (J. S.-S.).

Table 1 summarises the feedback received from the teachers on the TAKE10!^®^ model. Teachers from all three first-grade classrooms were surveyed in a post-intervention focus group. The principal of the school also participated in the focus group along with the school's technology and pedagogy expert and their curriculum expert. The teachers deemed that the children showed a desire to participate in more physical activity and found them to be more focused and ready to learn. In addition, the teachers found that they were more inclined to participate in physical activity themselves in order to be able to model positive behaviour for the children. The principal complaint about the programme was that the physical space available to implement the activities was very limited as the classrooms were very small.

**Table 1.** Questions and responses from the final focus group meeting with the teachers at Luis Matte Larraín public school (10 December 2014)

Why were you unable to implement the three daily sessions as proposed for the programme?	We only implemented two daily sessions. It was considered to be too difficult to fit in the three sessions as our schedule is already overcommitted with the amount of material that we must cover in only 5 h whereas most schools operate on a 7 h day
What were the easiest parts of the programme to implement? (Referring to the sequential stages for each activity including an initial relaxation, exercise, songs, stories, and final relaxation)	The sequence of each activity seemed to be adequate to the level and routine of the class. It allowed for the children to relax, be more focused, and ready to respond to questions like it were natural
Was there a part of the programme that was more difficult to implement (calming the children initially and getting their attention, the initial routine, the exercises, the songs, the stories, making do with the limited space, or the final relaxation)?	It was unanimously agreed among the teachers that the lack of space was the most difficult aspect of implementing the programme. The lack of space reduced the level of exercise intensity and some exercises had to be adapted to ensure proper ventilation and to avoid accidents
Do you believe that this is an effective way to teach positive physical activity habits to your children? Did you observe that the children changed their habits? If not, do you think you would see changes in their physical activity habits after a year of implementing this programme?	The programme was sufficient. The children were more active after participating, and we found them to be calmer in the classroom. A longer period of implementing the programme would have more impact
What benefits for you, the teachers, did you see with the implementation of TAKE10!? For those of you who were sedentary, did you find it a challenge to have to jump, run and move?	The teachers had to be models for the children's active participation. J. S. served as a model for the teachers to take on the routines

This initial programme included ninety-five students in three classrooms whose parents gave informed consent. There were six students who declined to participate for various reasons: five for health contraindications and one who was not attending classes. Parents of those six children were not asked informed consent.

Of the eighty-nine students studied, fifty-four were male and thirty-five were female, with an average age of 6·44 (sd 0·39, range 5·82–8·00) years. Table 2 presents data for observed changes both pre- and post-intervention for anthropometry, blood pressure and hand grip strength. Height measurements increased in a discrete manner during the intervention period; that increase would correspond to normal linear growth in children. The other two variables that were modified were systolic blood pressure, which decreased by an average of 2·5 mmHg, and waist circumference, which decreased by an average of 7·8 percentage points. However, none of the latter two comparisons that had significant *P* values in Table 2 had a power value of >0·80. Anthropometry and hand grip strength were not modified.

**Table 2.** Anthropometric characteristics of eighty-nine first-grade students in the Municipality of Puente Alto in Santiago, Chile, 2014 (Mean values and standard deviations; numbers and percentages)

	Pre-TAKE10!^®^		Post-TAKE10!^®^			
	Mean	sd	Mean	sd	*P*	Power
Weight (kg)	25·1	4·5	25·7	4·6	0·678	<40
Height (cm)	124·3	4·6	125·6	4·7	<0·001	99·9
BMI (kg/m^2^)	16·9	7·0	16·2	2·2	0·365	<40
BMI *Z*-score	0·1	1·3	0·1	1·2	0·450	<40
Nutritional status					0·413	<20
Underweight						
*n*	7		5			
%	7·0		5·6			
Normal weight						
*n*	58		62			
%	65·2		69·7			
Overweight						
*n*	15		14			
%	16·9		15·7			
Obese						
*n*	9		8			
%	10·1		9·0			
Waist circumference (cm)	59·2	6·3	59·2	5·8	0·152	<40
High waist circumference					0·016	<20
*n*	22		15			
%	24·7		16·9			
Triceps skin folds (mm)	10·8	4·4	11	4·6	0·666	<40
Subscapular skin folds (mm)	9·1	8·0	8·6	5·3	0·510	<40
Body fat percentage	18·2	8·0	17·9	7·3	0·438	<40
Systolic blood pressure (mmHg)	96·7	8·2	94·2	8·5	0·025	<40
Diastolic blood pressure (mmHg)	48·4	8·1	47·9	6·1	0·816	<40
High blood pressure					0·625	<20
*n*	3		1			
%	3·3		1·1			
Dynamometry (kg)	10·2	5·0	10·3	2·1	0·835	<40

The latter calculations and the lack of a control group are showing the weaknesses of this pilot study and that further research with a larger sample size and an experimental design is strongly needed. The implementation of the intervention should also be tested after the 3-month implementation phase, without the presence of supervisors.

Previous work evaluating the implementation of this programme in various other countries has shown an increase in physical activity levels in children^(^^18^^)^, an increase in energy expenditure by 25–35 kcal (105–146 kJ) per activity^(^^19^^)^, and improvements in concentration allowing children to be calmer and more focused in class^(^^20^^,^^21^^)^. A recent implementation of this programme in Brazil showed other favourable changes: children were more likely to select healthy food options and demonstrate positive physical activity behaviours^(^^22^^)^.

Other studies performed with different methods to the ones tested here have also shown positive results^(^^23^^–^^25^^)^ but, as with our study, a much longer time-frame of implementation would make evaluation of the results more valuable.

Other limitations of the study include the lack of a more precise measurement tool to record changes in behaviour and attitudes in the children. Previous randomised controlled studies included in a systematic review had physical activity objectively measured with accelerometers^(^^26^^)^.
