# Physical activity in the classroom to prevent childhood obesity: A pilot study in Santiago, Chile – CORRIGENDUM

**DOI:** 10.1017/jns.2017.32

**Published:** 2017-06-22

In the above mentioned article^(^^1^^)^ the authors were given in the wrong order. The correct order should have been

Pilar Arnaiz, Johana Soto-Sánchez, Juana Saavedra, Angélica Domínguez, Jaime Rozowski, Laura Iriarte, Jennifer Cantwell Wood and Francisco Mardones.

Additionally, Johana Soto-Sánchez's affiliation was given incorrectly, it should have read:

^3^ Department of Physical Education, Faculty of Physical and Sport Sciences, Universidad de Playa Ancha, Chile.
